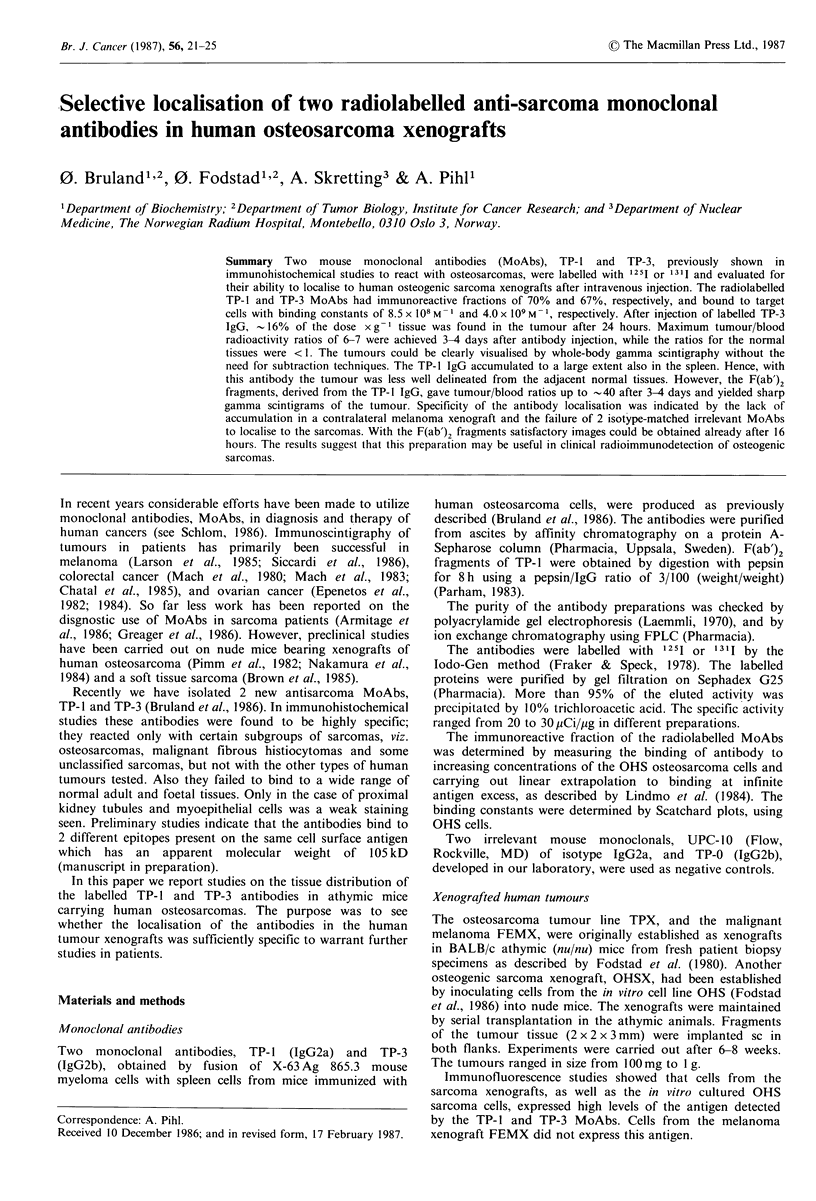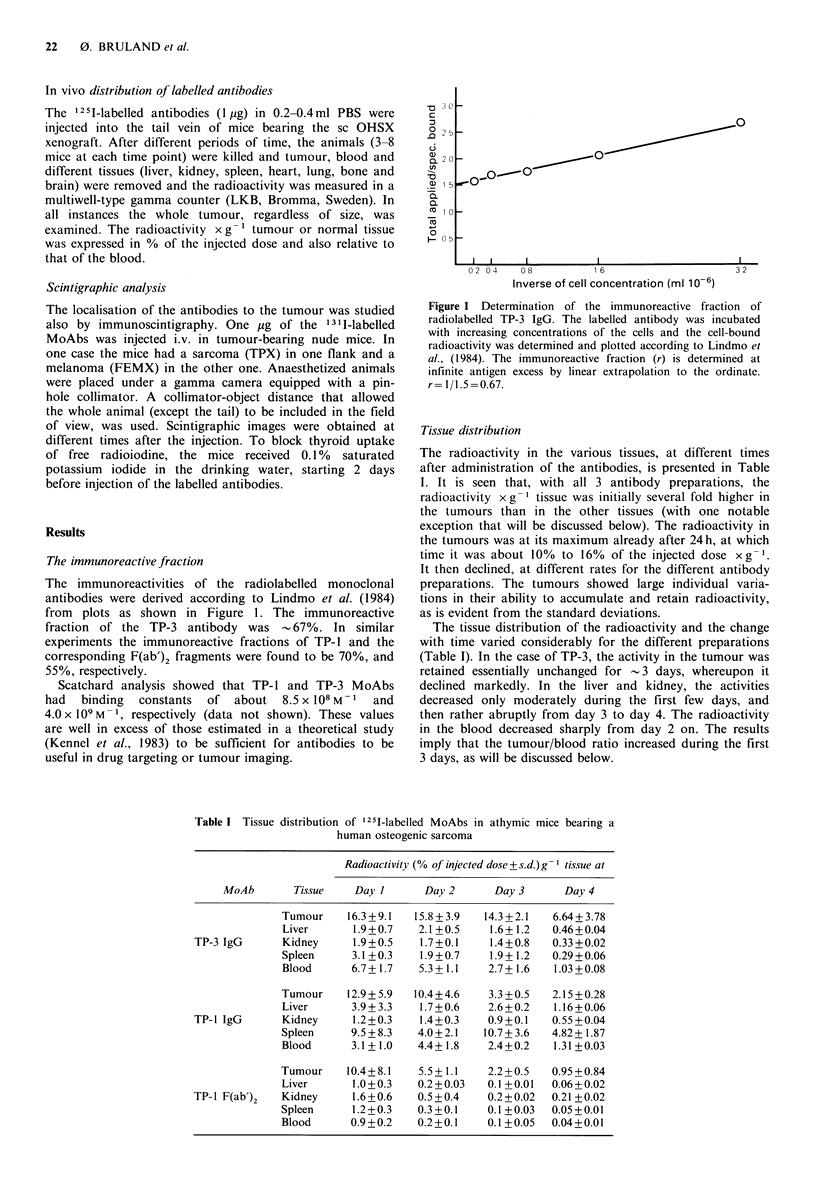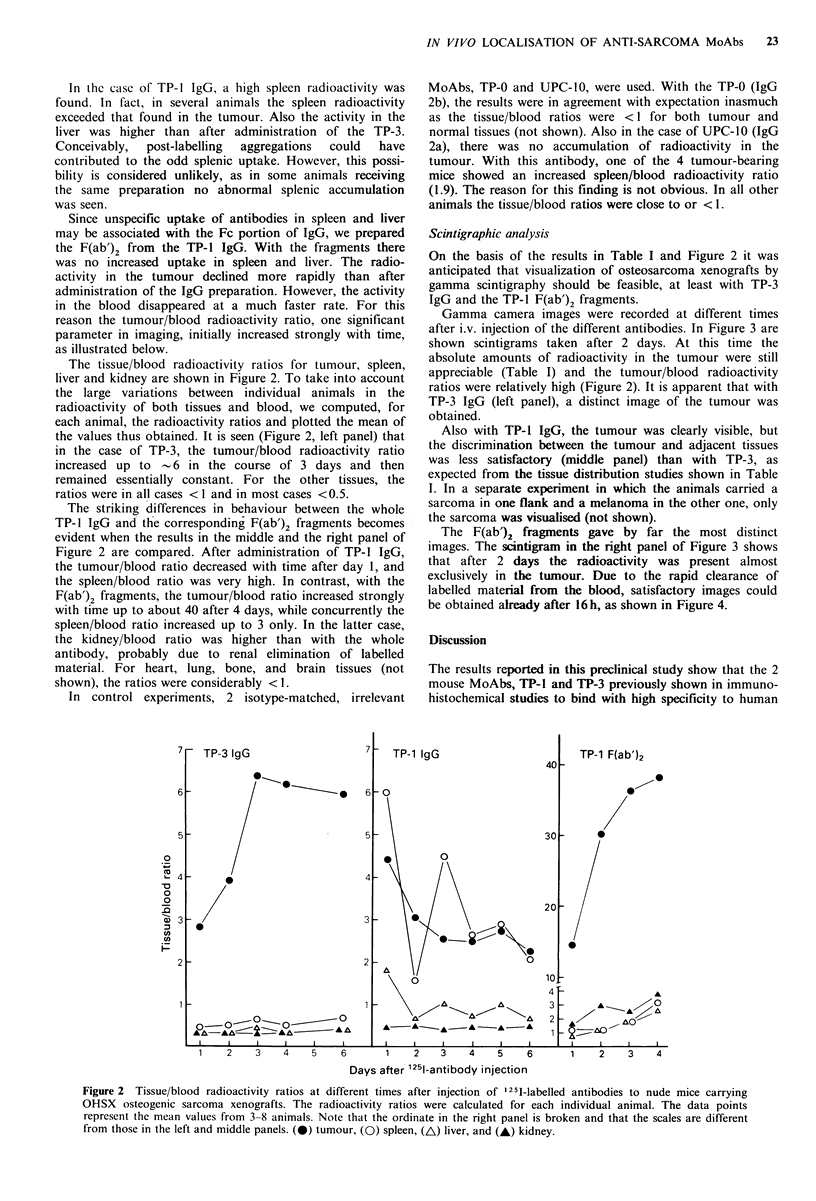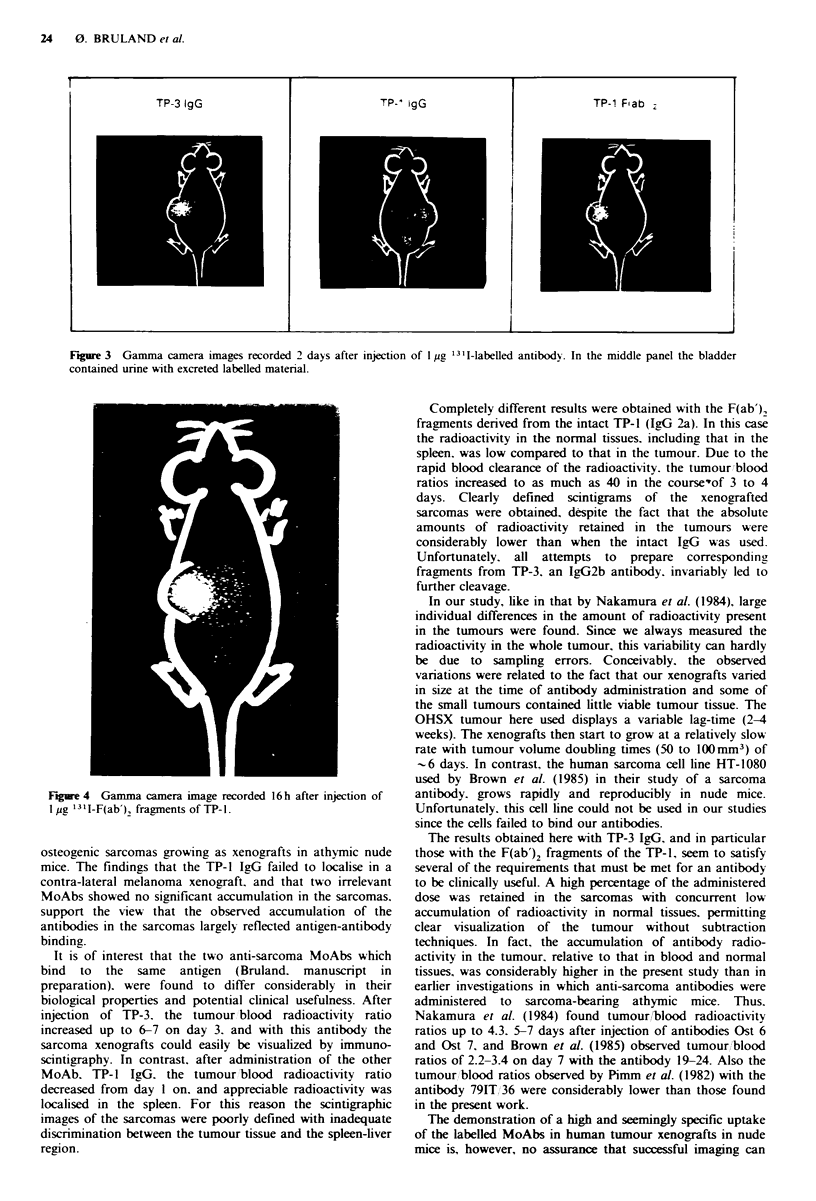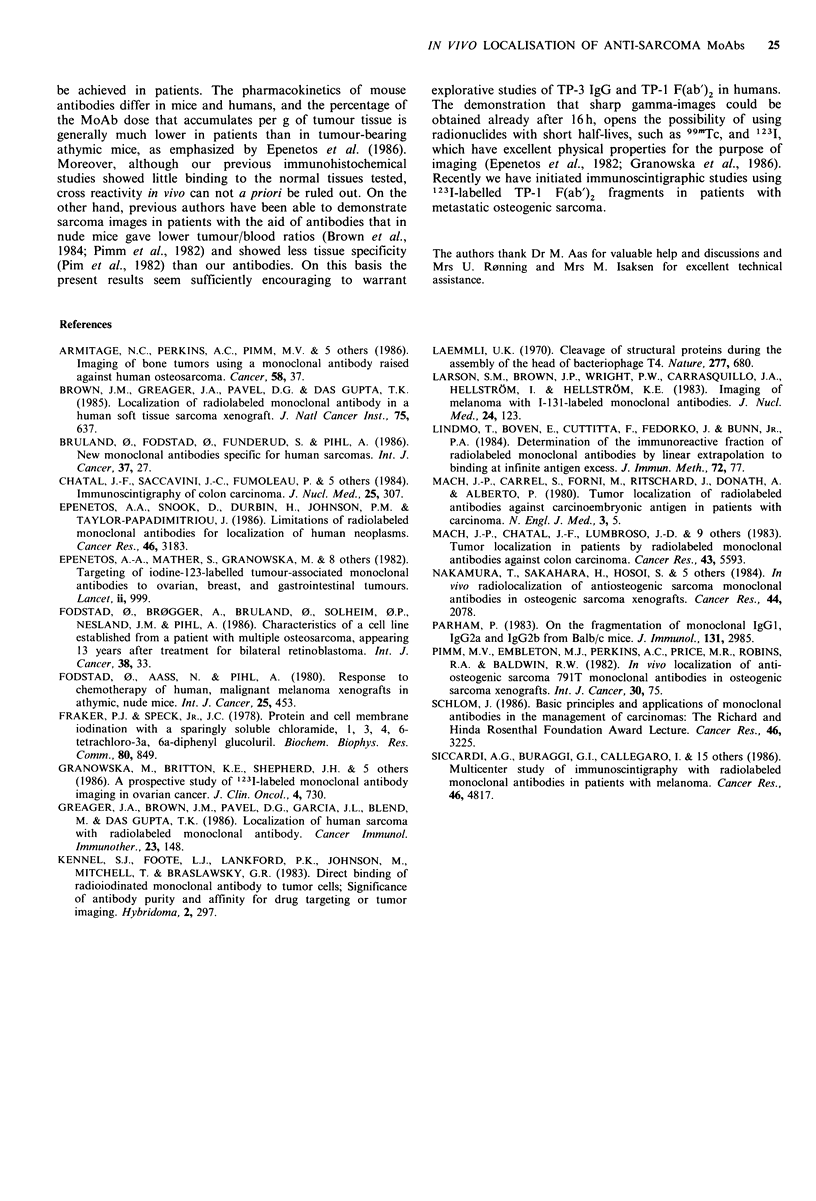# Selective localisation of two radiolabelled anti-sarcoma monoclonal antibodies in human osteosarcoma xenografts.

**DOI:** 10.1038/bjc.1987.146

**Published:** 1987-07

**Authors:** O. Bruland, O. Fodstad, A. Skretting, A. Pihl

## Abstract

Two mouse monoclonal antibodies (MoAbs), TP-1 and TP-3, previously shown in immunohistochemical studies to react with osteosarcomas, were labelled with 125I or 131I and evaluated for their ability to localise to human osteogenic sarcoma xenografts after intravenous injection. The radiolabelled TP-1 and TP-3 MoAbs had immunoreactive fractions of 70% and 67%, respectively, and bound to target cells with binding constants of 8.5 X 10(8) M-1 and 4.0 X 10(9) M-1, respectively. After injection of labelled TP-3 IgG, approximately 16% of the dose X g-1 tissue was found in the tumour after 24 hours. Maximum tumour/blood radioactivity ratios of 6-7 were achieved 3-4 days after antibody injection, while the ratios for the normal tissues were less than 1. The tumours could be clearly visualised by whole-body gamma scintigraphy without the need for subtraction techniques. The TP-1 IgG accumulated to a large extent also in the spleen. Hence, with this antibody the tumour was less well delineated from the adjacent normal tissues. However, the F(ab')2 fragments, derived from the TP-1 IgG, gave tumour/blood ratios up to approximately 40 after 3-4 days and yielded sharp gamma scintigrams of the tumour. Specificity of the antibody localisation was indicated by the lack of accumulation in a contralateral melanoma xenograft and the failure of 2 isotype-matched irrelevant MoAbs to localise to the sarcomas. With the F(ab')2 fragments satisfactory images could be obtained already after 16 hours. The results suggest that this preparation may be useful in clinical radioimmunodetection of osteogenic sarcomas.


*Br. J. Cancer* (1987), **56**, 21–25 © The Macmillan Press Ltd., 1987

### Selective localisation of two radiolabelled anti-sarcoma monoclonal antibodies in human osteosarcoma xenografts

Ø. Bruland[1,2], Ø. Fodstad[1,2], A. Skretting[3] & A. Pihl[1]

[1]*Department of Biochemistry;* [2]*Department of Tumor Biology, Institute for Cancer Research; and* [3]*Department of Nuclear Medicine, The Norwegian Radium Hospital, Montebello, 0310 Oslo 3, Norway.*

**Summary** Two mouse monoclonal antibodies (MoAbs), TP-1 and TP-3, previously shown in immunohistochemical studies to react with osteosarcomas, were labelled with $^{125}I$ or $^{131}I$ and evaluated for their ability to localise to human osteogenic sarcoma xenografts after intravenous injection. The radiolabelled TP-1 and TP-3 MoAbs had immunoreactive fractions of 70% and 67%, respectively, and bound to target cells with binding constants of $8.5 \times 10^8 M^{-1}$ and $4.0 \times 10^9 M^{-1}$, respectively. After injection of labelled TP-3 IgG, ~16% of the dose $\times g^{-1}$ tissue was found in the tumour after 24 hours. Maximum tumour/blood radioactivity ratios of 6–7 were achieved 3–4 days after antibody injection, while the ratios for the normal tissues were <1. The tumours could be clearly visualised by whole-body gamma scintigraphy without the need for subtraction techniques. The TP-1 IgG accumulated to a large extent also in the spleen. Hence, with this antibody the tumour was less well delineated from the adjacent normal tissues. However, the $F(ab')_2$ fragments, derived from the TP-1 IgG, gave tumour/blood ratios up to ~40 after 3–4 days and yielded sharp gamma scintigrams of the tumour. Specificity of the antibody localisation was indicated by the lack of accumulation in a contralateral melanoma xenograft and the failure of 2 isotype-matched irrelevant MoAbs to localise to the sarcomas. With the $F(ab')_2$ fragments satisfactory images could be obtained already after 16 hours. The results suggest that this preparation may be useful in clinical radioimmunodetection of osteogenic sarcomas.

In recent years considerable efforts have been made to utilize monoclonal antibodies, MoAbs, in diagnosis and therapy of human cancers (see Schlom, 1986). Immunoscintigraphy of tumours in patients has primarily been successful in melanoma (Larson *et al.*, 1985; Siccardi *et al.*, 1986), colorectal cancer (Mach *et al.*, 1980; Mach *et al.*, 1983; Chatal *et al.*, 1985), and ovarian cancer (Epenetos *et al.*, 1982; 1984). So far less work has been reported on the disgnostic use of MoAbs in sarcoma patients (Armitage *et al.*, 1986; Greager *et al.*, 1986). However, preclinical studies have been carried out on nude mice bearing xenografts of human osteosarcoma (Pimm *et al.*, 1982; Nakamura *et al.*, 1984) and a soft tissue sarcoma (Brown *et al.*, 1985).

Recently we have isolated 2 new antisarcoma MoAbs, TP-1 and TP-3 (Bruland *et al.*, 1986). In immunohistochemical studies these antibodies were found to be highly specific; they reacted only with certain subgroups of sarcomas, *viz.* osteosarcomas, malignant fibrous histiocytomas and some unclassified sarcomas, but not with the other types of human tumours tested. Also they failed to bind to a wide range of normal adult and foetal tissues. Only in the case of proximal kidney tubules and myoepithelial cells was a weak staining seen. Preliminary studies indicate that the antibodies bind to 2 different epitopes present on the same cell surface antigen which has an apparent molecular weight of 105 kD (manuscript in preparation).

In this paper we report studies on the tissue distribution of the labelled TP-1 and TP-3 antibodies in athymic mice carrying human osteosarcomas. The purpose was to see whether the localisation of the antibodies in the human tumour xenografts was sufficiently specific to warrant further studies in patients.

### Materials and methods

### *Monoclonal antibodies*

Two monoclonal antibodies, TP-1 (IgG2a) and TP-3 (IgG2b), obtained by fusion of X-63 Ag 865.3 mouse myeloma cells with spleen cells from mice immunized with human osteosarcoma cells, were produced as previously described (Bruland *et al.*, 1986). The antibodies were purified from ascites by affinity chromatography on a protein A-Sepharose column (Pharmacia, Uppsala, Sweden). $F(ab')_2$ fragments of TP-1 were obtained by digestion with pepsin for 8 h using a pepsin/IgG ratio of 3/100 (weight/weight) (Parham, 1983).

The purity of the antibody preparations was checked by polyacrylamide gel electrophoresis (Laemmli, 1970), and by ion exchange chromatography using FPLC (Pharmacia).

The antibodies were labelled with $^{125}I$ or $^{131}I$ by the Iodo-Gen method (Fraker & Speck, 1978). The labelled proteins were purified by gel filtration on Sephadex G25 (Pharmacia). More than 95% of the eluted activity was precipitated by 10% trichloroacetic acid. The specific activity ranged from 20 to 30 $\mu$Ci/$\mu$g in different preparations.

The immunoreactive fraction of the radiolabelled MoAbs was determined by measuring the binding of antibody to increasing concentrations of the OHS osteosarcoma cells and carrying out linear extrapolation to binding at infinite antigen excess, as described by Lindmo *et al.* (1984). The binding constants were determined by Scatchard plots, using OHS cells.

Two irrelevant mouse monoclonals, UPC-10 (Flow, Rockville, MD) of isotype IgG2a, and TP-0 (IgG2b), developed in our laboratory, were used as negative controls.

### *Xenografted human tumours*

The osteosarcoma tumour line TPX, and the malignant melanoma FEMX, were originally established as xenografts in BALB/c athymic (*nu*/*nu*) mice from fresh patient biopsy specimens as described by Fodstad *et al.* (1980). Another osteogenic sarcoma xenograft, OHSX, had been established by inoculating cells from the *in vitro* cell line OHS (Fodstad *et al.*, 1986) into nude mice. The xenografts were maintained by serial transplantation in the athymic animals. Fragments of the tumour tissue (2 × 2 × 3 mm) were implanted sc in both flanks. Experiments were carried out after 6–8 weeks. The tumours ranged in size from 100 mg to 1 g.

Immunofluorescence studies showed that cells from the sarcoma xenografts, as well as the *in vitro* cultured OHS sarcoma cells, expressed high levels of the antigen detected by the TP-1 and TP-3 MoAbs. Cells from the melanoma xenograft FEMX did not express this antigen.

Correspondence: A. Pihl.
Received 10 December 1986; and in revised form, 17 February 1987.

*In vivo distribution of labelled antibodies*

The $^{125}$I-labelled antibodies (1 $\mu$g) in 0.2–0.4 ml PBS were injected into the tail vein of mice bearing the sc OHSX xenograft. After different periods of time, the animals (3–8 mice at each time point) were killed and tumour, blood and different tissues (liver, kidney, spleen, heart, lung, bone and brain) were removed and the radioactivity was measured in a multiwell-type gamma counter (LKB, Bromma, Sweden). In all instances the whole tumour, regardless of size, was examined. The radioactivity $\times g^{-1}$ tumour or normal tissue was expressed in % of the injected dose and also relative to that of the blood.

*Scintigraphic analysis*

The localisation of the antibodies to the tumour was studied also by immunoscintigraphy. One $\mu$g of the $^{131}$I-labelled MoAbs was injected i.v. in tumour-bearing nude mice. In one case the mice had a sarcoma (TPX) in one flank and a melanoma (FEMX) in the other one. Anaesthetized animals were placed under a gamma camera equipped with a pinhole collimator. A collimator-object distance that allowed the whole animal (except the tail) to be included in the field of view, was used. Scintigraphic images were obtained at different times after the injection. To block thyroid uptake of free radioiodine, the mice received 0.1% saturated potassium iodide in the drinking water, starting 2 days before injection of the labelled antibodies.

### Results

*The immunoreactive fraction*

The immunoreactivities of the radiolabelled monoclonal antibodies were derived according to Lindmo *et al.* (1984) from plots as shown in Figure 1. The immunoreactive fraction of the TP-3 antibody was ~67%. In similar experiments the immunoreactive fractions of TP-1 and the corresponding F(ab')$_2$ fragments were found to be 70%, and 55%, respectively.

Scatchard analysis showed that TP-1 and TP-3 MoAbs had binding constants of about $8.5\times10^8\,M^{-1}$ and $4.0\times10^9\,M^{-1}$, respectively (data not shown). These values are well in excess of those estimated in a theoretical study (Kennel *et al.*, 1983) to be sufficient for antibodies to be useful in drug targeting or tumour imaging.

**Figure 1** Determination of the immunoreactive fraction of radiolabelled TP-3 IgG. The labelled antibody was incubated with increasing concentrations of the cells and the cell-bound radioactivity was determined and plotted according to Lindmo *et al.*, (1984). The immunoreactive fraction ($r$) is determined at infinite antigen excess by linear extrapolation to the ordinate. $r=1/1.5=0.67$.

*Tissue distribution*

The radioactivity in the various tissues, at different times after administration of the antibodies, is presented in Table I. It is seen that, with all 3 antibody preparations, the radioactivity $\times g^{-1}$ tissue was initially several fold higher in the tumours than in the other tissues (with one notable exception that will be discussed below). The radioactivity in the tumours was at its maximum already after 24 h, at which time it was about 10% to 16% of the injected dose $\times g^{-1}$. It then declined, at different rates for the different antibody preparations. The tumours showed large individual variations in their ability to accumulate and retain radioactivity, as is evident from the standard deviations.

The tissue distribution of the radioactivity and the change with time varied considerably for the different preparations (Table I). In the case of TP-3, the activity in the tumour was retained essentially unchanged for ~3 days, whereupon it declined markedly. In the liver and kidney, the activities decreased only moderately during the first few days, and then rather abruptly from day 3 to day 4. The radioactivity in the blood decreased sharply from day 2 on. The results imply that the tumour/blood ratio increased during the first 3 days, as will be discussed below.

**Table I** Tissue distribution of $^{125}$I-labelled MoAbs in athymic mice bearing a human osteogenic sarcoma

| | | *Radioactivity (% of injected dose ± s.d.) g$^{-1}$ tissue at* | | | |
|---|---|---|---|---|---|
| *MoAb* | *Tissue* | *Day 1* | *Day 2* | *Day 3* | *Day 4* |
| TP-3 IgG | Tumour | 16.3±9.1 | 15.8±3.9 | 14.3±2.1 | 6.64±3.78 |
| | Liver | 1.9±0.7 | 2.1±0.5 | 1.6±1.2 | 0.46±0.04 |
| | Kidney | 1.9±0.5 | 1.7±0.1 | 1.4±0.8 | 0.33±0.02 |
| | Spleen | 3.1±0.3 | 1.9±0.7 | 1.9±1.2 | 0.29±0.06 |
| | Blood | 6.7±1.7 | 5.3±1.1 | 2.7±1.6 | 1.03±0.08 |
| TP-1 IgG | Tumour | 12.9±5.9 | 10.4±4.6 | 3.3±0.5 | 2.15±0.28 |
| | Liver | 3.9±3.3 | 1.7±0.6 | 2.6±0.2 | 1.16±0.06 |
| | Kidney | 1.2±0.3 | 1.4±0.3 | 0.9±0.1 | 0.55±0.04 |
| | Spleen | 9.5±8.3 | 4.0±2.1 | 10.7±3.6 | 4.82±1.87 |
| | Blood | 3.1±1.0 | 4.4±1.8 | 2.4±0.2 | 1.31±0.03 |
| TP-1 F(ab')$_2$ | Tumour | 10.4±8.1 | 5.5±1.1 | 2.2±0.5 | 0.95±0.84 |
| | Liver | 1.0±0.3 | 0.2±0.03 | 0.1±0.01 | 0.06±0.02 |
| | Kidney | 1.6±0.6 | 0.5±0.4 | 0.2±0.02 | 0.21±0.02 |
| | Spleen | 1.2±0.3 | 0.3±0.1 | 0.1±0.03 | 0.05±0.01 |
| | Blood | 0.9±0.2 | 0.2±0.1 | 0.1±0.05 | 0.04±0.01 |

In the case of TP-1 IgG, a high spleen radioactivity was found. In fact, in several animals the spleen radioactivity exceeded that found in the tumour. Also the activity in the liver was higher than after administration of the TP-3. Conceivably, post-labelling aggregations could have contributed to the odd splenic uptake. However, this possibility is considered unlikely, as in some animals receiving the same preparation no abnormal splenic accumulation was seen.

Since unspecific uptake of antibodies in spleen and liver may be associated with the Fc portion of IgG, we prepared the $F(ab')_2$ from the TP-1 IgG. With the fragments there was no increased uptake in spleen and liver. The radioactivity in the tumour declined more rapidly than after administration of the IgG preparation. However, the activity in the blood disappeared at a much faster rate. For this reason the tumour/blood radioactivity ratio, one significant parameter in imaging, initially increased strongly with time, as illustrated below.

The tissue/blood radioactivity ratios for tumour, spleen, liver and kidney are shown in Figure 2. To take into account the large variations between individual animals in the radioactivity of both tissues and blood, we computed, for each animal, the radioactivity ratios and plotted the mean of the values thus obtained. It is seen (Figure 2, left panel) that in the case of TP-3, the tumour/blood radioactivity ratio increased up to $\sim 6$ in the course of 3 days and then remained essentially constant. For the other tissues, the ratios were in all cases $<1$ and in most cases $<0.5$.

The striking differences in behaviour between the whole TP-1 IgG and the corresponding $F(ab')_2$ fragments becomes evident when the results in the middle and the right panel of Figure 2 are compared. After administration of TP-1 IgG, the tumour/blood ratio decreased with time after day 1, and the spleen/blood ratio was very high. In contrast, with the $F(ab')_2$ fragments, the tumour/blood ratio increased strongly with time up to about 40 after 4 days, while concurrently the spleen/blood ratio increased up to 3 only. In the latter case, the kidney/blood ratio was higher than with the whole antibody, probably due to renal elimination of labelled material. For heart, lung, bone, and brain tissues (not shown), the ratios were considerably $<1$.

In control experiments, 2 isotype-matched, irrelevant MoAbs, TP-0 and UPC-10, were used. With the TP-0 (IgG 2b), the results were in agreement with expectation inasmuch as the tissue/blood ratios were $<1$ for both tumour and normal tissues (not shown). Also in the case of UPC-10 (IgG 2a), there was no accumulation of radioactivity in the tumour. With this antibody, one of the 4 tumour-bearing mice showed an increased spleen/blood radioactivity ratio (1.9). The reason for this finding is not obvious. In all other animals the tissue/blood ratios were close to or $<1$.

*Scintigraphic analysis*

On the basis of the results in Table I and Figure 2 it was anticipated that visualization of osteosarcoma xenografts by gamma scintigraphy should be feasible, at least with TP-3 IgG and the TP-1 $F(ab')_2$ fragments.

Gamma camera images were recorded at different times after i.v. injection of the different antibodies. In Figure 3 are shown scintigrams taken after 2 days. At this time the absolute amounts of radioactivity in the tumour were still appreciable (Table I) and the tumour/blood radioactivity ratios were relatively high (Figure 2). It is apparent that with TP-3 IgG (left panel), a distinct image of the tumour was obtained.

Also with TP-1 IgG, the tumour was clearly visible, but the discrimination between the tumour and adjacent tissues was less satisfactory (middle panel) than with TP-3, as expected from the tissue distribution studies shown in Table I. In a separate experiment in which the animals carried a sarcoma in one flank and a melanoma in the other one, only the sarcoma was visualised (not shown).

The $F(ab')_2$ fragments gave by far the most distinct images. The scintigram in the right panel of Figure 3 shows that after 2 days the radioactivity was present almost exclusively in the tumour. Due to the rapid clearance of labelled material from the blood, satisfactory images could be obtained already after 16 h, as shown in Figure 4.

### Discussion

The results reported in this preclinical study show that the 2 mouse MoAbs, TP-1 and TP-3 previously shown in immunohistochemical studies to bind with high specificity to human

**Figure 3** Gamma camera images recorded 2 days after injection of 1 $\mu g$ $^{131}I$-labelled antibody. In the middle panel the bladder contained urine with excreted labelled material.

**Figure 4** Gamma camera image recorded 16 h after injection of 1 $\mu g$ $^{131}I$-$F(ab')_2$ fragments of TP-1.

osteogenic sarcomas growing as xenografts in athymic nude mice. The findings that the TP-1 IgG failed to localise in a contra-lateral melanoma xenograft, and that two irrelevant MoAbs showed no significant accumulation in the sarcomas, support the view that the observed accumulation of the antibodies in the sarcomas largely reflected antigen-antibody binding.

It is of interest that the two anti-sarcoma MoAbs which bind to the same antigen (Bruland, manuscript in preparation), were found to differ considerably in their biological properties and potential clinical usefulness. After injection of TP-3, the tumour/blood radioactivity ratio increased up to 6–7 on day 3, and with this antibody the sarcoma xenografts could easily be visualized by immunoscintigraphy. In contrast, after administration of the other MoAb, TP-1 IgG, the tumour/blood radioactivity ratio decreased from day 1 on, and appreciable radioactivity was localised in the spleen. For this reason the scintigraphic images of the sarcomas were poorly defined with inadequate discrimination between the tumour tissue and the spleen-liver region.

Completely different results were obtained with the $F(ab')_2$ fragments derived from the intact TP-1 (IgG 2a). In this case the radioactivity in the normal tissues, including that in the spleen, was low compared to that in the tumour. Due to the rapid blood clearance of the radioactivity, the tumour/blood ratios increased to as much as 40 in the course of 3 to 4 days. Clearly defined scintigrams of the xenografted sarcomas were obtained, despite the fact that the absolute amounts of radioactivity retained in the tumours were considerably lower than when the intact IgG was used. Unfortunately, all attempts to prepare corresponding fragments from TP-3, an IgG2b antibody, invariably led to further cleavage.

In our study, like in that by Nakamura *et al.* (1984), large individual differences in the amount of radioactivity present in the tumours were found. Since we always measured the radioactivity in the whole tumour, this variability can hardly be due to sampling errors. Conceivably, the observed variations were related to the fact that our xenografts varied in size at the time of antibody administration and some of the small tumours contained little viable tumour tissue. The OHSX tumour here used displays a variable lag-time (2–4 weeks). The xenografts then start to grow at a relatively slow rate with tumour volume doubling times (50 to 100 $mm^3$) of ~6 days. In contrast, the human sarcoma cell line HT-1080 used by Brown *et al.* (1985) in their study of a sarcoma antibody, grows rapidly and reproducibly in nude mice. Unfortunately, this cell line could not be used in our studies since the cells failed to bind our antibodies.

The results obtained here with TP-3 IgG, and in particular those with the $F(ab')_2$ fragments of the TP-1, seem to satisfy several of the requirements that must be met for an antibody to be clinically useful. A high percentage of the administered dose was retained in the sarcomas with concurrent low accumulation of radioactivity in normal tissues, permitting clear visualization of the tumour without subtraction techniques. In fact, the accumulation of antibody radioactivity in the tumour, relative to that in blood and normal tissues, was considerably higher in the present study than in earlier investigations in which anti-sarcoma antibodies were administered to sarcoma-bearing athymic mice. Thus, Nakamura *et al.* (1984) found tumour/blood radioactivity ratios up to 4.3, 5–7 days after injection of antibodies Ost 6 and Ost 7, and Brown *et al.* (1985) observed tumour/blood ratios of 2.2–3.4 on day 7 with the antibody 19–24. Also the tumour/blood ratios observed by Pimm *et al.* (1982) with the antibody 791T/36 were considerably lower than those found in the present work.

The demonstration of a high and seemingly specific uptake of the labelled MoAbs in human tumour xenografts in nude mice is, however, no assurance that successful imaging can be achieved in patients. The pharmacokinetics of mouse antibodies differ in mice and humans, and the percentage of the MoAb dose that accumulates per g of tumour tissue is generally much lower in patients than in tumour-bearing athymic mice, as emphasized by Epenetos *et al.* (1986). Moreover, although our previous immunohistochemical studies showed little binding to the normal tissues tested, cross reactivity *in vivo* can not *a priori* be ruled out. On the other hand, previous authors have been able to demonstrate sarcoma images in patients with the aid of antibodies that in nude mice gave lower tumour/blood ratios (Brown *et al.*, 1984; Pimm *et al.*, 1982) and showed less tissue specificity (Pim *et al.*, 1982) than our antibodies. On this basis the present results seem sufficiently encouraging to warrant explorative studies of TP-3 IgG and TP-1 $F(ab')_2$ in humans. The demonstration that sharp gamma-images could be obtained already after 16 h, opens the possibility of using radionuclides with short half-lives, such as $^{99m}Tc$, and $^{123}I$, which have excellent physical properties for the purpose of imaging (Epenetos *et al.*, 1982; Granowska *et al.*, 1986). Recently we have initiated immunoscintigraphic studies using $^{123}I$-labelled TP-1 $F(ab')_2$ fragments in patients with metastatic osteogenic sarcoma.

The authors thank Dr M. Aas for valuable help and discussions and Mrs U. Rønning and Mrs M. Isaksen for excellent technical assistance.

### References

ARMITAGE, N.C., PERKINS, A.C., PIMM, M.V. & 5 others (1986). Imaging of bone tumors using a monoclonal antibody raised against human osteosarcoma. *Cancer*, **58**, 37.

BROWN, J.M., GREAGER, J.A., PAVEL, D.G. & DAS GUPTA, T.K. (1985). Localization of radiolabeled monoclonal antibody in a human soft tissue sarcoma xenograft. *J. Natl Cancer Inst.*, **75**, 637.

BRULAND, Ø., FODSTAD, Ø., FUNDERUD, S. & PIHL, A. (1986). New monoclonal antibodies specific for human sarcomas. *Int. J. Cancer*, **37**, 27.

CHATAL, J.-F., SACCAVINI, J.-C., FUMOLEAU, P. & 5 others (1984). Immunoscintigraphy of colon carcinoma. *J. Nucl. Med.*, **25**, 307.

EPENETOS, A.A., SNOOK, D., DURBIN, H., JOHNSON, P.M. & TAYLOR-PAPADIMITRIOU, J. (1986). Limitations of radiolabeled monoclonal antibodies for localization of human neoplasms. *Cancer Res.*, **46**, 3183.

EPENETOS, A.-A., MATHER, S., GRANOWSKA, M. & 8 others (1982). Targeting of iodine-123-labelled tumour-associated monoclonal antibodies to ovarian, breast, and gastrointestinal tumours. *Lancet*, **ii**, 999.

FODSTAD, Ø., BRØGGER, A., BRULAND, Ø., SOLHEIM, Ø.P., NESLAND, J.M. & PIHL, A. (1986). Characteristics of a cell line established from a patient with multiple osteosarcoma, appearing 13 years after treatment for bilateral retinoblastoma. *Int. J. Cancer*, **38**, 33.

FODSTAD, Ø., AASS, N. & PIHL, A. (1980). Response to chemotherapy of human, malignant melanoma xenografts in athymic, nude mice. *Int. J. Cancer*, **25**, 453.

FRAKER, P.J. & SPECK, JR., J.C. (1978). Protein and cell membrane iodination with a sparingly soluble chloramide, 1, 3, 4, 6-tetrachloro-3a, 6a-diphenyl glucoluril. *Biochem. Biophys. Res. Comm.*, **80**, 849.

GRANOWSKA, M., BRITTON, K.E., SHEPHERD, J.H. & 5 others (1986). A prospective study of $^{123}I$-labeled monoclonal antibody imaging in ovarian cancer. *J. Clin. Oncol.*, **4**, 730.

GREAGER, J.A., BROWN, J.M., PAVEL, D.G., GARCIA, J.L., BLEND, M. & DAS GUPTA, T.K. (1986). Localization of human sarcoma with radiolabeled monoclonal antibody. *Cancer Immunol. Immunother.*, **23**, 148.

KENNEL, S.J., FOOTE, L.J., LANKFORD, P.K., JOHNSON, M., MITCHELL, T. & BRASLAWSKY, G.R. (1983). Direct binding of radioiodinated monoclonal antibody to tumor cells; Significance of antibody purity and affinity for drug targeting or tumor imaging. *Hybridoma*, **2**, 297.

LAEMMLI, U.K. (1970). Cleavage of structural proteins during the assembly of the head of bacteriophage T4. *Nature*, **277**, 680.

LARSON, S.M., BROWN, J.P., WRIGHT, P.W., CARRASQUILLO, J.A., HELLSTRÖM, I. & HELLSTRÖM, K.E. (1983). Imaging of melanoma with I-131-labeled monoclonal antibodies. *J. Nucl. Med.*, **24**, 123.

LINDMO, T., BOVEN, E., CUTTITTA, F., FEDORKO, J. & BUNN, JR., P.A. (1984). Determination of the immunoreactive fraction of radiolabeled monoclonal antibodies by linear extrapolation to binding at infinite antigen excess. *J. Immun. Meth.*, **72**, 77.

MACH, J.-P., CARREL, S., FORNI, M., RITSCHARD, J., DONATH, A. & ALBERTO, P. (1980). Tumor localization of radiolabeled antibodies against carcinoembryonic antigen in patients with carcinoma. *N. Engl. J. Med.*, **3**, 5.

MACH, J.-P., CHATAL, J.-F., LUMBROSO, J.-D. & 9 others (1983). Tumor localization in patients by radiolabeled monoclonal antibodies against colon carcinoma. *Cancer Res.*, **43**, 5593.

NAKAMURA, T., SAKAHARA, H., HOSOI, S. & 5 others (1984). *In vivo* radiolocalization of antiosteogenic sarcoma monoclonal antibodies in osteogenic sarcoma xenografts. *Cancer Res.*, **44**, 2078.

PARHAM, P. (1983). On the fragmentation of monoclonal IgG1, IgG2a and IgG2b from Balb/c mice. *J. Immunol.*, **131**, 2985.

PIMM, M.V., EMBLETON, M.J., PERKINS, A.C., PRICE, M.R., ROBINS, R.A. & BALDWIN, R.W. (1982). *In vivo* localization of anti-osteogenic sarcoma 791T monoclonal antibodies in osteogenic sarcoma xenografts. *Int. J. Cancer*, **30**, 75.

SCHLOM, J. (1986). Basic principles and applications of monoclonal antibodies in the management of carcinomas: The Richard and Hinda Rosenthal Foundation Award Lecture. *Cancer Res.*, **46**, 3225.

SICCARDI, A.G., BURAGGI, G.I., CALLEGARO, I. & 15 others (1986). Multicenter study of immunoscintigraphy with radiolabeled monoclonal antibodies in patients with melanoma. *Cancer Res.*, **46**, 4817.